# Trends in anticoagulant prescribing: a review of local policies in English primary care

**DOI:** 10.1186/s12913-020-5058-1

**Published:** 2020-04-03

**Authors:** Katherine H. Ho, Maria van Hove, Gillian Leng

**Affiliations:** 1 National Institute for Health and Care Excellence, 10 Spring Gardens, London, SW1A 2BU UK; 2 Harvard Global Health Institute, Harvard University, 42 Church St, Cambridge, MA 02138 USA

**Keywords:** DOAC, NOAC, warfarin, anticoagulant, atrial fibrillation, prescribing, stroke, primary care, local policy, England

## Abstract

**Background:**

Oral anticoagulants are prescribed for stroke prophylaxis in patients with atrial fibrillation, which is the most common heart arrhythmia worldwide. The vitamin K antagonist (VKA) warfarin is a long-established anticoagulant. However, newer direct oral anticoagulants (DOACs) have been recently introduced as an alternative. Given the prevalence of atrial fibrillation, anticoagulant choice has substantial clinical and financial implications for healthcare systems. In this study, we explore trends and geographic variation in anticoagulant prescribing in English primary care. Because national guidelines in England do not specify a first-line anticoagulant, we investigate the association between local policies and prescribing data.

**Methods:**

Primary care prescribing data of anticoagulants for all NHS practices from 2014 to 2019 in England was obtained from the ePACT2 database. Public formularies were accessed online to obtain local anticoagulation prescribing policies for 89.5% of clinical commissioning groups (CCGs). These were categorized according to their recommendations: no local policies, warfarin as first-line, or identification of a preferred DOAC (but not a preferred anticoagulant). Local policies were cross-tabulated with pooled prescribing data to measure the strength of association with Cramér’s V.

**Results:**

Nationally, prescribing of DOACs increased from 9% of all anticoagulants in 2014 to 74% in 2019, while that of warfarin declined accordingly. Still, there was significant local variation. Across geographical regions, DOACs ranged from 53 to 99% of all anticoagulants. Most CCGs (73%) did not specify a first-line choice, and 16% recommended warfarin first line. Only 11% designated a preferred DOAC. Policies with a preferred DOAC indeed correlated with increased prescribing of that DOAC (Cramér’s V = 0.25, 0.27, 0.38 for rivaroxaban, apixaban, edoxaban respectively). However, local policies showed a negligible relationship with the classes of anticoagulants prescribed—DOAC or VKA (Cramér’s V = 0.01).

**Conclusions:**

Nationally, the use of DOACs to treat atrial fibrillation has increased rapidly. Despite this, significant geographical variation in uptake remains. This study provides insights on how local policies relate to this variation. Our findings suggest that, in the absence of a nationally recommended first-line anticoagulant, local prescribing policies may aid in deciding between individual DOACs, but not in adjudicating between DOACs and vitamin K antagonists (i.e. warfarin) as general classes.

## Background

Atrial fibrillation (AF) impacts millions of individuals worldwide and is the most common heart arrhythmia. Individuals with AF have an increased risk of stroke, which inflicts a heavy burden on both individuals and societies [1, 2]. Anticoagulants are now recognized as an important treatment in the prophylaxis of stroke due to AF.

Warfarin is a long-established anticoagulant which requires close blood monitoring and dietary modifications. It is part of a class of drugs called Vitamin K antagonists (VKAs) that inhibit the enzyme Vitamin K epoxide reductase. In the past decade, however, new direct oral anticoagulants (DOACs) have become available. They operate by a different cellular mechanism, instead inhibiting Factor Xa or thrombin. Randomized controlled trials have shown that DOACs are non-inferior to warfarin when used for stroke prevention [3–5], and some analyses of clinical effectiveness suggest they are actually preferable [6]. DOACs are also convenient due to a lack of mandatory monitoring, and—compared to warfarin—have fewer problematic food and drug interactions.

In England, there is considerable effort at the national level to coordinate and disseminate information on medical best practices. For example, the National Institute for Health and Care Excellence (NICE)—with which the authors are affiliated—convenes panels of physicians, statisticians, and patient representatives to discuss scientific literature. Ultimately, NICE is responsible for conducting appraisals of new medicines to decide whether they represent a cost-effective use of resources for the National Health Service (NHS). These assessments of value also consider clinical efficacy. Only drugs with a positive appraisal are routinely commissioned—and therefore used—in the English NHS.

Currently, four DOACs have received positive appraisals: apixaban, rivaroxaban, dabigatran, and edoxaban. Thus, the NHS is legally obligated to pay for these medications.

In practice though, local implementation may lag behind the national green light. There are several barriers to the actual uptake of these newly approved medicines. DOACs have been deemed by NICE cost-effective in the long term, and independent analyses have concluded that DOACs are superior to warfarin in this respect [6]. But the initial cost of a prescription for a DOAC is much higher than that of warfarin, which may give pause to local commissioning agencies. Also, while several real-world studies on DOACs have established reassuring safety profiles [7, 8], fears of increased bleeding linger, particularly in the media [9]. There existed no antidote for rivaroxaban or apixaban until the introduction of andexanet alfa in 2018—and still, no antidote has been recommended by a NICE technology appraisal. This is all in addition to potential inertia derived from prescriber preference and experience level [10]. Finally, a bevy of local factors such as pharmaceutical rebates, local incentives and policies, and patient demographics influence prescribing choices.

In addition to its decision-making capacity, NICE also issues guidelines on management of diseases and conditions, which may indicate which treatment should be considered first line. However, NICE guidelines on AF management do not give a clear indication in this respect. The guidelines do not indicate whether vitamin K antagonists or DOACs should be preferred. Moreover, they do not specify which DOAC should be preferred out of the class. Accordingly, there is likely to be significant variation in the prescribing practices of anticoagulants nationally between different areas.

This study aims to determine trends in use of anticoagulants. In particular, we examine geographical variation and the impact of local policies on anticoagulant prescribing choices for the management of atrial fibrillation. We also assess whether local policies are a barrier to the uptake of DOACs.

## Methods

### Review of anticoagulant policies

#### Policy categories

Formulary websites for every Clinical Commissioning Group (CCG) in England were accessed online in July and August 2019. There were 191 active CCGs at time of review; each CCG is an official body of the NHS that coordinates and commissions healthcare services for the local community. Criteria were used by one of the authors (KHH) to classify each CCG’s policy into one of five categories:

- No local policy preference given (“No”)
- Recommendation for warfarin as an overall first-line anticoagulant (“Warfarin”)
- Identification of a preferred DOAC (“Apixaban,” “Rivaroxaban,” or “Edoxaban,” respectively), but not necessarily preference for DOACs overall

For the last category, we did not include CCGs that indicated a preferred DOAC, but recommended warfarin as the overall first-line anticoagulant, putting these instead under the “Warfarin” category. This last category did not necessarily require policies to prefer DOACs as a class over warfarin. We chose this more inclusive method of categorization because no CCGs indicated an explicit preference for a DOAC as an absolute overall first-line anticoagulant over warfarin.

#### Criteria for categorization

Local policies were categorized using a strict set of requirements to reduce subjectivity. The format of the formularies was conducive to this approach, as policies tended to be clearly flagged using certain phrases.

1. We assigned a category only to policies that were publically accessible online in a CCG’s official primary care formulary.

We required that polices were either directly written on the formulary webpage, or included in policy documents linked on the webpage. The formulary for each CCG was identified by Google searches or browsing on the CCG website. In a few ambiguous cases, confirmation was obtained directly from the CCG through freedom of information requests.

We chose to focus on public formularies to maintain consistency while maximizing the CCGs included in the study. Moreover, CCGs often integrate these public formularies into the clinical information systems used in primary care, which can display real-time suggestions of preferred medications to guide decision-making by prescribers [11].

2. We looked for the words “first line,” “preferred,” or “recommended” to describe anticoagulant use, or anticoagulation for atrial fibrillation. If a policy existed but none of these phrases were used, we categorized the policy as having “No” recommendation.
3. We excluded and did not categorize CCGs that relied only on “traffic light” lists, or CCGs that had no formularies altogether. “Traffic light” lists only provided guidance on prescribing responsibilities, such as whether a medication could be initiated in primary care or secondary care.
4. We did not categorize recommendations that were specified to apply only to hospitals (secondary care) or for other indications such as deep-vein thrombosis.
5. We only considered information on the formulary to be a recommendation if one or two, but not three or more anticoagulants were singled out. If a policy existed but recommended three or more anticoagulants, we categorized it as having “No” recommendation.

### Secondary analysis of prescribing data

#### Data source

The data included all prescriptions of apixaban, rivaroxaban, edoxaban, dabigatran, and warfarin written by NHS general practices in England from January 2014 to August 2019. This information was published by the NHS Business Services Authority in the ePACT2 database and was accessed in November 2019. The data was aggregated at the CCG level on a monthly basis.

#### Statistical analysis

We used descriptive statistics to examine national trends and variation in anticoagulant use, and to present a graph of policies.

To analyze the association between prescribing policy and actual outcome, we cross-tabulated prescribing policy and ePACT2 anticoagulant prescribing data. We constructed tables that compared local policies against prescriptions measured in Daily Defined Doses (DDD). This is a standardized amount of medication defined by the WHO to be the average maintenance dose per day for an adult [12]. In this case, the DDD were calibrated based on an indication of AF.

This data was all analyzed with chi-square analysis, which yielded *p* values that indicated the statistical significance of any differences. To assay not just statistical significance but practical relevance, we then used Cramér’s V to assess the strength of association between policies and actual prescribing data.

## Results

**Fig. 1:**
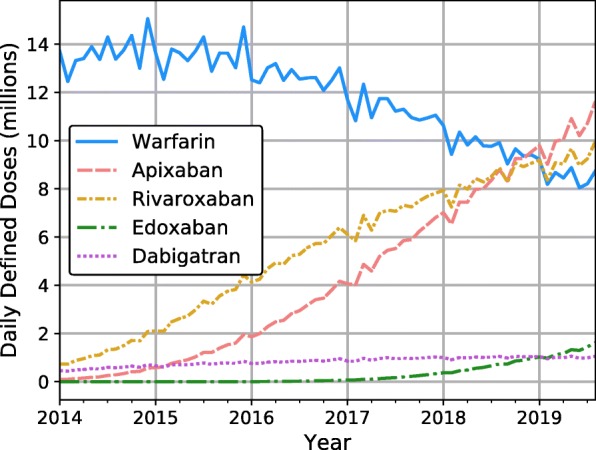
National trends in anticoagulant prescribing, Jan 2014 – Aug 2019

Nationally, prescribing of DOACs steadily increased from 9% of all anticoagulants in 2014 to 74% in 2019. That of warfarin declined from 91 to 26%. Figure 1 shows these national trends measured in millions of DDD in each month-long reporting period from January 2014 to August 2019. Apixaban and rivaroxaban were the most commonly used DOACs, with 11.6 million and 10.0 million doses respectively in the last reporting month, compared with warfarin’s 8.7 million. Prescribing for dabigatran was relatively constant around 1 million DDD per month. Edoxaban prescribing was low, but climbed steadily starting in early 2017, representing an entry point later than the other anticoagulants.

Overall, total anticoagulant prescribing nearly doubled during the period examined, from 15.0 million doses in January 2014 to 33.0 million doses in August 2019.

**Fig. 2:**
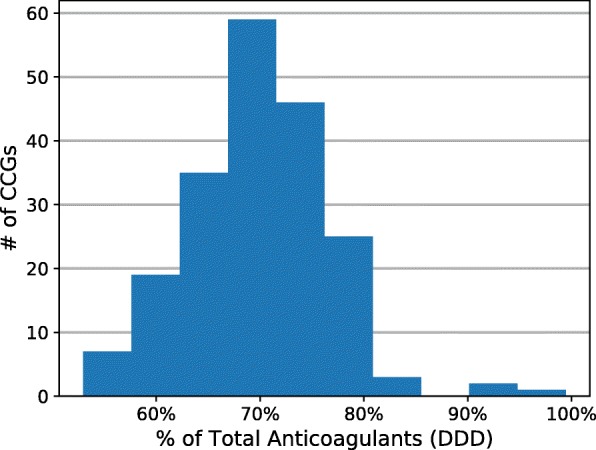
Variation in uptake of DOACs across CCGs. For the last 12 months of data available, Sept 2018 – Aug 2019. CCGs with less than 150 total DDD were excluded

Despite a clear national shift toward DOACs, a different picture emerged when examining prescribing in individual CCGs. Figure 2 shows a histogram of the percentage DOACs out of total anticoagulants at the CCG level. We used only the last year of prescribing data accessed to reflect more current practices in anticoagulation. While the Blackpool CCG only used DOACs for 53.0% of anticoagulant prescriptions, this proportion reached as high as 99.5% in the Heywood, Middleton, and Rochdale CCG in the time period examined. The mean was 69.8% (SD = 6.6%).

To study possible reasons for the variation in DOAC use, we looked at local policies. Policy categories were assigned to 171 CCGs, which represented 89.5% of recently active reporting CCGs during July and August 2019. Reporting CCGs included any CCG that submitted data on anticoagulant items during this time. The number of unique reporting CCGs from the entire period Jan 2014 – August 2019 was much higher, at 221. This included now-defunct CCGs that since merged into entities listed under other names. The 10.5% we excluded were CCGs where we were unable to find an official online formulary, often because that CCG did not use a formulary.

The review revealed that, of 171 CCGs classified, most (72.5%) did not specify any prescribing recommendations (“No”). Of the 47 CCGs that did specify recommendations, the majority (59.6%) recommended warfarin as an overall first-line treatment for anticoagulation (Table 1). The next most common category was “Rivaroxaban,” which represented 17.0% of all recommendations and 8 CCGs. This was followed by “Edoxaban” with 12.8% of recommendations (6 CCGs) and “Apixaban” with 10.6% of recommendations (5 CCGs). Note that here we refer to categories defined previously for the purposes of this study. For example, “Rivaroxaban” denotes CCGs that specified rivaroxaban as a first-line DOAC, but not necessarily as an overall first-line anticoagulant.

**Table 1:** Distribution of anticoagulant policies adopted by CCGs

Recommendation	Number of CCGs
No	124
Warfarin	28
Rivaroxaban	8
Edoxaban	6
Apixaban	5
Total	171

For policies accessed July – Aug 2019

**Fig. 3:**
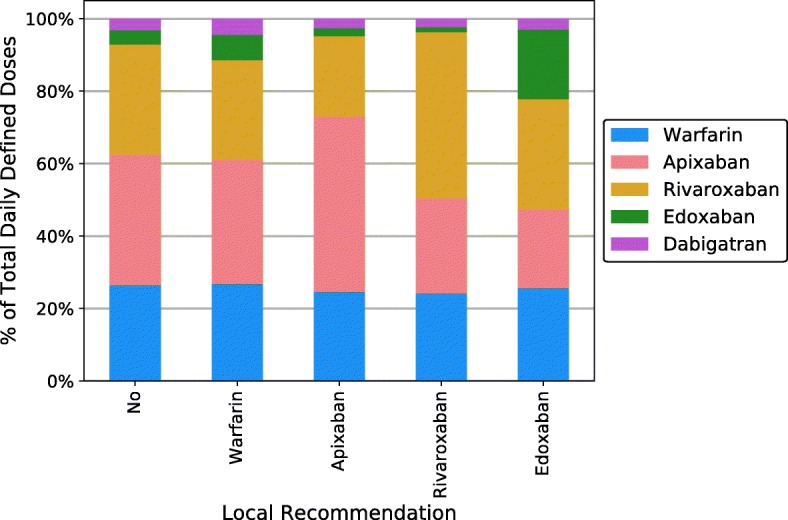
Association between policies and prescribing practices, July – Aug 2019

We then studied the correlation between local policies, as categorized above, and actual prescribing data. This comparison is shown graphically in Fig. 3. Each column represents a category of policies, and the colored bars above show pooled prescribing data in all CCGs bearing that policy from July to August 2019. The policy categorizations and prescribing data came from the same two-month time period.

We made two types of comparisons:

1. Prescriptions for warfarin vs. DOACs, for local policy categories “No” vs. “Warfarin” vs. any DOAC (“Edoxaban” + “Rivaroxaban” + “Apixaban”) (3 × 2 table)
2. Prescriptions for apixaban vs. all other DOACs, for local policy categories “Apixaban” vs. all other DOACs (“Edoxaban” + “Rivaroxaban”) (2 × 2 table)
  - Repeated considering rivaroxaban
  - Repeated considering edoxaban

The results of the first comparison are shown in Table 2. There was a statistically significant difference in choice between warfarin and DOACs depending on whether the local CCG had no recommendation (“No”), recommended warfarin outright (“Warfarin”), or specified a preferred DOAC (“Apixaban,” “Rivaroxaban,” “Edoxaban,” pooled). The *p* value was < 0.005. Cramér’s V for this 3 × 2 table was 0.013, representing a negligible effect size. Cramér’s V ranges from 0 to 1 with larger values showing a stronger association between variables, and corrects for sample size differences. Graphically, the blue bar representing warfarin prescriptions was relatively constant between all five categories (Fig. 3).

**Table 2:** Association between local prescribing policy and prescribing of warfarin vs. DOACs

	Prescriptions (DDD) (millions)		*p* value	Cramér’s V
Local policy	Warfarin	DOACs^b^		
No	11.154	31.092	< 0.005	0.013
Warfarin	2.431	6.644		
DOAC^a^	1.374	4.205		

For policies accessed July – Aug 2019, and prescribing data spanning the same period

^a^Includes the categories “Apixaban,” “Rivaroxaban,” “Edoxaban”

^b^Includes prescriptions of all DOACs: apixaban, rivaroxaban, edoxaban, and dabigatran

Table 3 shows the results of statistical analysis preformed on three separate 2 × 2 contingency tables. Each 2 × 2 table corresponds to a comparison involving rivaroxaban, apixaban, or edoxaban. The purpose of each comparison was to ascertain whether recommending an individual DOAC actually correlated with prescribing preference over alternatives within the same class. For example, rivaroxaban prescriptions were compared to non-rivaroxaban DOAC prescriptions (denoted by the “Other DOAC” column) under two conditions: first in CCGs that recommended rivaroxaban (“Rivaroxaban”), and second in CCGs that recommended another DOAC (“Edoxaban” or “Apixaban”, denoted by the “Other DOAC” row). This was repeated for apixaban and edoxaban. Each 2 × 2 table investigates whether prescribing of a given DOAC meaningfully differs between CCGs either recommending it or not.

**Table 3:** Association between local DOAC preference and prescribing of DOACs

		Prescriptions (DDD) (millions)		*p* value	Cramér’s V
Local DOAC Policy		Recommended DOAC	Other DOAC^b^		
Rivaroxaban	Rivaroxaban	1.291	0.843	< 0.005	0.249
	Other DOAC^a^	0.738	1.333		
Apixaban	Apixaban	0.609	0.339	< 0.005	0.268
	Other DOAC^a^	1.068	2.189		
Edoxaban	Edoxaban	0.290	0.833	< 0.005	0.377
	Other DOAC^a^	0.066	3.016		

For policies accessed July – Aug 2019, and prescribing data spanning the same period

^a^Includes the two other categories from the set {“Rivaroxaban,” “Apixaban,” “Edoxaban”} besides the one being considered

^b^Includes pooled prescriptions of three DOACs from the set {rivaroxaban, apixaban, edoxaban, dabigatran} besides the one being considered

All three *p* values obtained were very small, less than < 0.005. However, in contrast to Table 2, there were moderately high values for Cramer’s V. For rivaroxaban, apixaban, and edoxaban, we obtained 0.249, 0.268, and 0.377 respectively. This denoted a moderate association between policy and prescribing when looking at individual DOAC choice, with the highest association found for edoxaban.

## Discussion

Stroke is costly for both individuals and societies. In 2016, 5.5 million people worldwide died from a stroke [13]. Of survivors, approximately 20% are unable to walk without full physical assistance [14], and 20% experience language impairments [15]. In the U.K., health and social care spending amounted to £46,039 per patient in the 5 years post-stroke [16].

Encouraging proper anticoagulation therapy is thus an essential cornerstone of preventive strategy. Given the importance of anticoagulation in public health, this study attempted to characterize and investigate trends in prescribing at both a local and national level. Nationally, use of DOACs increased dramatically from 9 to 74% of anticoagulants in just 5 years, while prescriptions for warfarin fell. This trend could suggest a substitution effect wherein patients on warfarin are switched to DOACs. However, there was also near-doubling of total anticoagulant prescriptions from 2014 to 2019. This suggests that the increase in DOACs could also be driven by an initiation of new patients on the medication, not just switching of existing patients. This is consistent with findings that rate of DOAC initiation has increased in recent years compared to a drop in initiation of VKAs [17].

Certainly, the choice between DOACs and VKAs is an area of active clinical research [3–5, 7, 8, 18]. However, to translate any research findings into real-world implementation, it is crucial to understand the variety of factors that drive local decision-making. There was wide variation in trends at the local level, with DOAC uptake ranging from 53.0 to 99.4% in different CCGs. Although patient demographics must play a role in this discrepancy, countless other factors could be at play. For example, knowledge of favorable clinical trial results involving DOACs could sway physician preferences. There may be local financial incentives for either DOAC prescription [19] or warfarin monitoring [20], and pharmaceutical companies may provide local rebates that make certain anticoagulants much more cost-effective [21]. There may be carryover from secondary care. Since NICE does not recommend any anticoagulant as first-line, local recommendations may influence prescribers.

We characterized this last correlation between local policies and local prescribing practices. There was negligible correlation (Cramér’s V = 0.013) between type of local policy (no recommendation, warfarin first-line, or identification of a preferred DOAC) and class of anticoagulant prescriptions (DOAC or VKA). However, within the category of DOACs, local policies showed a moderate association with choice of individual DOAC. The Cramér’s V value was 0.249, 0.268, and 0.377 for rivaroxaban, apixaban, and edoxaban respectively.

There were significant *p* values for all comparisons. However, a large number of observations—DDD values in the millions—mean even very small differences will yield p values suggesting statistical significance. In this case, measures of strength of association, such as Cramér’s V, are more practically relevant [22].

The results suggest that local anticoagulation recommendations do not influence choices between DOACs and VKAs, but play an important role in determining choice of specific DOAC.

This interpretation makes sense because DOACs and VKAs are very different, while individual DOACs are more similar to each other. For example, warfarin interacts with a number of foods and medications, including cranberries, alcohol, many antibiotics, and ibuprofen. DOACs have few interactions. Warfarin requires routine blood monitoring, while DOACs do not. Only warfarin is recommended for the treatment of patients with valvular atrial fibrillation, defined as the presence of moderate-to-severe mitral stenosis or a mechanical heart valve [23, 24]. The upfront cost of the two drugs is vastly different. Under drug tariff prices in the British National Formulary published by NICE and accessed in October 2019, 4 weeks of the DDD for warfarin cost £1.28. Meanwhile, the cost for 4 weeks of apixaban, rivaroxaban, edoxaban, or dabigatran was £53.20, £50.40, £49.00, or £47.60, respectively.

These differences between DOACs and VKAs impact financing, patient choice, and medical considerations, which may be the main determinants in prescribing choices rather than local policy recommendations. For instance, a Canadian survey showed that food-drug interactions were the single most important attribute in patients’ anticoagulation preferences [25]. However, local recommendations that differentiate between the less-distinguishable DOACs may be perceived as more helpful by prescribers. This may be especially true in cases where the recommendations provide genuine insight into unique local conditions, such as a rebate received by the CCG that makes one DOAC comparatively cheaper. This is known to be the case for edoxaban wherein a manufacturer rebate program cuts the per-pack price by 29% [21]. This may account for the relatively strong association between policy and practice for edoxaban, for which we found a Cramér’s V value 0.109 greater than the next strongest association.

Although there were relatively strong associations between anticoagulant prescribing and certain policies where they existed, our review of these policies on public formularies revealed that 72.5% of CCGs did not have any such policies in place. This—along with the negligible association found between policies and prescribing of warfarin vs DOACs—suggests that local policies cannot fully explain the geographical variation in prescribing shown by Fig. 2, or the dramatic shift in anticoagulant use shown in Fig. 1.

Although existing studies have explored national trends and local variation in anticoagulation for patients with atrial fibrillation [7, 17, 26–28], this study represents the first comprehensive review of local anticoagulation policies in England. Other studies use dedicated primary care databanks that contain more patient-level data, while we use national NHS prescribing data aggregated by CCG. The ePACT2 database does not include indications. Still, previous studies in U.K. primary care found the majority of patients prescribed anticoagulants have an indication of AF rather than venous thromboembolism (VTE), the next most common indication [17]. Patients on anticoagulation for AF typically have a longer median treatment time than those with VTE [7], which means more doses. It is reasonable to assume that the main driver of anticoagulant doses in our data is AF. This lack of granular patient-level data represents a trade-off with the wide coverage of the data. Our review of policies was comprehensive, classifying 89.5% of currently active CCG that reported data during the time period studied.

However, a limitation is that our review of local policies only captured a snapshot at one point in time. Although we used prescribing data from July and August 2019 that roughly coincided with data collection on policies during the same period, we could not ensure that these policies remained in effect during the entire two-month period. Another limitation is that the study is purely observational. We can draw conclusions about correlation but not causation. Moreover, we could not differentiate between existing patients and new patients. Perhaps the association between policy and prescribing practice is driven by initiating many new patients on the recommended DOAC, but existing patients are not being switched. In this case, our results may simply be reflective of local AF screening campaigns that recruit large numbers of new patients. The finding that local policy correlates with DOAC choice may only translate over to cases where the number of new patients shows robust growth. This represents a barrier to uptake that policymakers should consider.

Our results indicate that in the majority of CCGs, local policies as they are currently implemented do not drive choice of anticoagulant for atrial fibrillation. Future research should aim to characterize the complex influence of other possible factors, including financial and structural features of local healthcare economies.

## Conclusions

In order to effectively encourage adoption of clinically robust, cost-efficient therapeutics, it is worthwhile for national policymakers and researchers to understand the complex factors driving local prescribing choices. This study investigates one such factor in the case of anticoagulation for atrial fibrillation: local policies. A review of policies in England and analysis of corresponding prescribing data revealed that local recommendations are indeed moderately correlated with choices between individual DOACs. However, they do not drive national trends or variation in prescribing of different classes of anticoagulants (DOAC vs VKA). Our study provides insight into the implementation of national healthcare initiatives in local systems.

## Data Availability

All data generated and analyzed in this study’s review of local policies and analysis of prescribing data is available from the corresponding author on reasonable request.

## Abbreviations

CCG: Clinical Commissioning Group
DDD: Defined daily dose
DOAC: Direct oral anticoagulant
ePACT2: Electronic Prescribing Analysis and Cost 2
NHS: National Health Service
NICE: National Institute for Health and Care Excellence
NOAC: Non-vitamin K antagonist oral anticoagulant
VKA: Vitamin K antagonist
